# A Review on the Assessment of Stress Conditions for Simultaneous Production of Microalgal Lipids and Carotenoids

**DOI:** 10.3389/fmicb.2016.00546

**Published:** 2016-05-03

**Authors:** Amritpreet K. Minhas, Peter Hodgson, Colin J. Barrow, Alok Adholeya

**Affiliations:** ^1^Biotechnology and Bioresources Division, TERI-Deakin Nanobiotechnology Centre, The Energy and Resources Institute, India Habitat CentreNew Delhi, India; ^2^Institute for Frontier Materials, Deakin UniversityVictoria, VIC, Australia; ^3^School of Life and Environmental Sciences, Deakin UniversityVictoria, VIC, Australia

**Keywords:** carotenoids, PUFA, autotrophic, heterotrophic, lipids, biorefinery

## Abstract

Microalgal species are potential resource of both biofuels and high-value metabolites, and their production is growth dependent. Growth parameters can be screened for the selection of novel microalgal species that produce molecules of interest. In this context our review confirms that, autotrophic and heterotrophic organisms have demonstrated a dual potential, namely the ability to produce lipids as well as value-added products (particularly carotenoids) under influence of various physico-chemical stresses on microalgae. Some species of microalgae can synthesize, besides some pigments, very-long-chain polyunsaturated fatty acids (VL-PUFA,>20C) such as docosahexaenoic acid and eicosapentaenoic acid, those have significant applications in food and health. Producing value-added by-products in addition to biofuels, fatty acid methyl esters (FAME), and lipids has the potential to improve microalgae-based biorefineries by employing either the autotrophic or the heterotrophic mode, which could be an offshoot of biotechnology. The review considers the potential of microalgae to produce a range of products and indicates future directions for developing suitable criteria for choosing novel isolates through bioprospecting large gene pool of microalga obtained from various habitats and climatic conditions.

## Introduction

Microalgae are autotrophic organism that consumes light energy and inorganic nutrients and produces biomass rich in value-added products such as lipids, carbohydrates, proteins, and pigments (Markou and Nerantzis, 2013). Highly explored for lipids, microalgae also produce metabolites such as carotenoids (lutein, zeaxanthin, and astaxanthin), long-chain polyunsaturated fatty acids (LC-PUFA), and vitamins (Pal et al., 2011), that are widely used in nutraceuticals industries as food additives (Priyadarshani and Rath, 2012). The interest for large scale cultivation of microalgal biomass that are rich reserves of high value metabolites is increasing, however detailed literature on its optimal and economically more sustainable production is not available (Clarens et al., 2010; Norsker et al., 2011; Soratana and Landis, 2011).

The production of lipids, carotenoids and algal biomass can be enhanced under environmental stress factors (Mata et al., 2010; Mulders et al., 2014; González et al., 2015). In addition to stress factors, selection and use of microalgal species and strains is also important for enhancing the metabolite production. Therefore, exploration of a wider and more diverse gene pool of microalgae is required. For economical production, deriving multiple products such as lipids and high-value by-products from the same biomass in one growth cycle is one way (Campenni et al., 2013; Nobre et al., 2013). Further optimizing culture conditions, by selecting organisms that can overcome the limitations imposed by ambient conditions, and by selecting strains that produce high lipid content can also lower the unit cost of microalgae-based biofuels (Wijffels et al., 2010).

Current research on microalgae based production is focused on developing autotrophic or heterotrophic cultivation strategies most conducive for production of lipids and other value-added products in laboratory-scale biorefineries. The present article provides a review on the cultivation strategies used for microalgae growth, mainly focusing on the environmental stress factors, for increasing the production of lipids and value-added products (namely, lutein, astaxanthin, and β-carotene). We provide information on developing methods that can maximize the production of biofuel, biomass, and other value-added by-products (carotenoids) from the same species or strains of microalgae that will become a win–win strategy in the coming decades. Some of these phenomenons are covered in this review, however for details on other metabolites such as phycobilins, polysaccharides and vitamins see review of Skjånes et al. (2013) and Markou and Nerantzis (2013).

## Microalgal lipid synthesis

Microalgal lipids are divided in to two main categories, namely those—used as biofuel (with 14–20 carbon chains) and as food (containing 20 carbon chains; Jacob-Lopes et al., 2015). Green algae share their ancestry with higher plants and their metabolic mechanisms and photosynthetic pigments are similar to those found in higher plants (Yu et al., 2011). Some microalgae also produce large amounts of lipids in the form of triacylglycerides (TAGs). The synthesis of lipids in microalgae varies with the species (from either freshwater or marine habitats or in cyanobacteria) used (Hu et al., 2008). Compared to cyanobacteria, two unique features of algae are the ability to store large quantities of lipids in the form of oil globules in parts of the cell other than chloroplasts and the ability to link the electron transport system to hydrogen production (Radakovits et al., 2010). Li et al. (2012) showed that accumulation of neutral lipids in the cell occurs through the conversion of either starch or carbon to lipids, but conversion depends on microalgal strains, different strains have different mechanism for transmitting the carbon flux from the carbohydrate pathway for synthesizing lipids.

**Figure 1:**
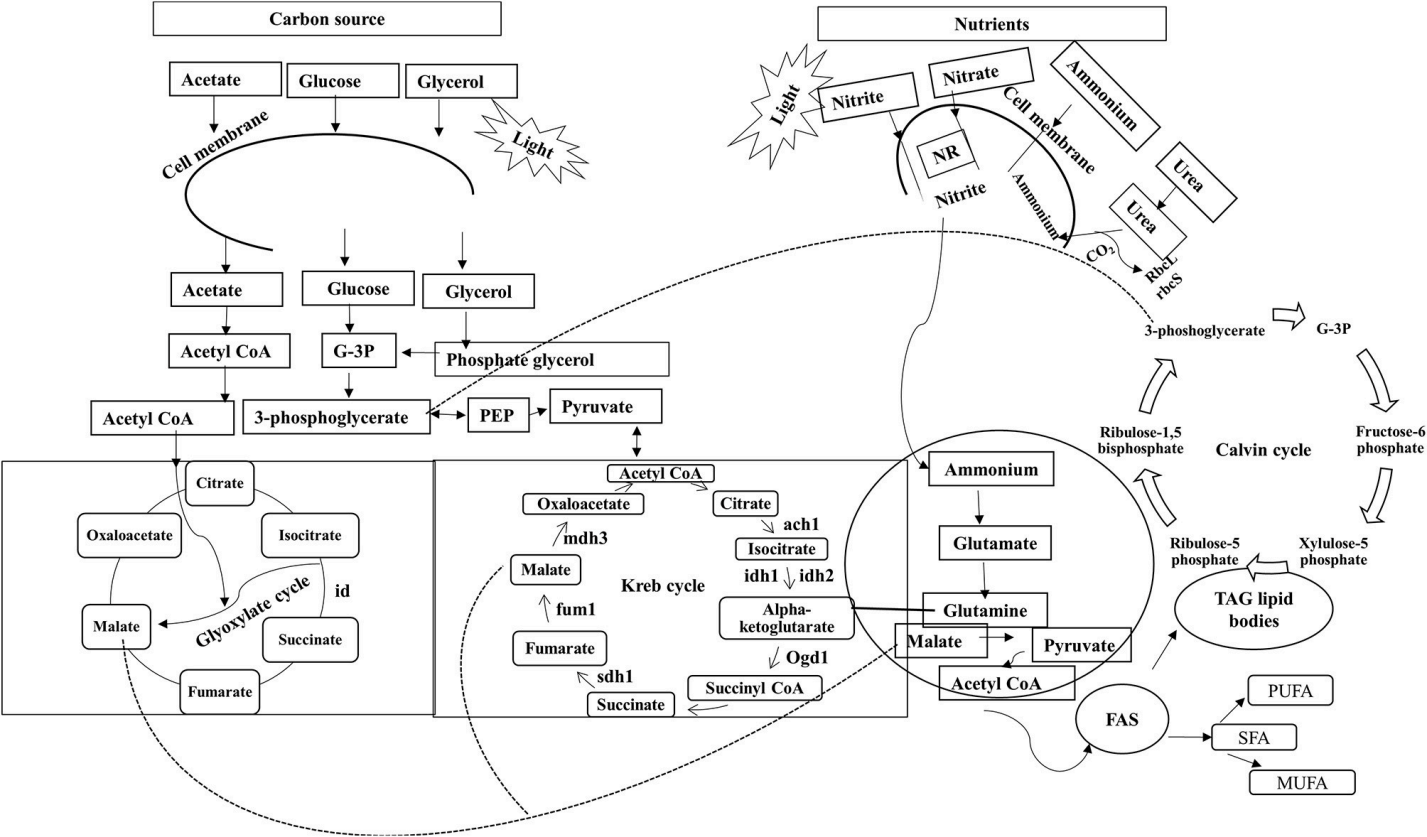
**Role of various carbon sources and nitrates in lipid biosynthesis**. Sn-Glycerol-3 phosphate NAD+oxidoreducatse; ach 1, aconitate; idh 1, isocitrate dehydrogenase-NADH dependent; idh 2, isocitrate dehydrogenase-NADPH dependent; *ogd* 1, ketoglutarate dehydrogenase; fum1, fumarate hydratase; sdh 1, succinate dehydrogenase; mdh3, malate dehydrogenase; *scla* 1, succinate-CoA ligase (ADP forming); FAS, fatty acid synthesis; TAG, triacylglycerols; id, isocitrate dehydrogenase;rbcL, Ribulose bisophosphate carboxylase/oxygenase large subunit; rbcS, Ribulose bisophosphate carboxylase/oxygenase small subunit; prk, Phosphoribulokinase.

In unicellular organisms, lipid biosynthesis follows a complex pathway where synthesis initiates with the formation of acetyl CoA by the ACCase gene through acetyl CoA carboxylation. This is the key step at which carbon is assigned for lipid synthesis (Hu et al., 2008), as shown in Figure 1 (adapted and modified from Perez-Garcia et al., 2011). Lipid biosynthesis has been explored most extensively in *Chlamydomonas reinhardtii* (Moellering and Benning, 2010). The first metabolic study for increasing the accumulation of FAs was reported in *Cyclotella cryptica*, in which the acetyl-CoA carboxylase gene (*ACCase*) was overexpressed (Dunahay et al., 1996). Some microalgae adapt well to various environmental conditions that affect cellular processes including lipid metabolism (Juneja et al., 2013). Microalgae are also known to synthesize very -long-chain polyunsaturated FAs (VL-PUFA, >20C), and the synthesis is regulated by desaturases and elongases, which are temperature-sensitive enzymes (Niu et al., 2013).

Polyunsaturated fatty acids (PUFA) are mainly found in fish originate from digested microalgae in the marine environment. Moreover, eicosapentaenoic acid (EPA) and docosahexaenoic acid (DHA) are the most valuable FAs found in microalgae and, their high content found in microalgae is rich in these two FAs (Spolaore et al., 2006; Yen et al., 2013), make it suitable for biofuel manufacturing (Gimpel et al., 2015). Under abiotic forms of stress, many microalgae produce TAGs that can serve as feedstock for biofuel production. However, the amounts of TAGs vary with the species and the genera (Trentacoste et al., 2013). Various stress factors induce changes in the metabolic activities of a cell such as activation of starch and accumulation of TAGs leading to accumulation of lipid bodies in algae (Johnson and Alric, 2013). Since lipid biosynthesis is a complex process, it not only presents greater challenges in increasing the production of specific lipids but also in increasing overall lipid production in microalgae. It is therefore important to increase the application of metabolic engineering to lipid biosynthesis.

## Strategies to enhance stress based lipids changes in microalgae

**Figure 2:**
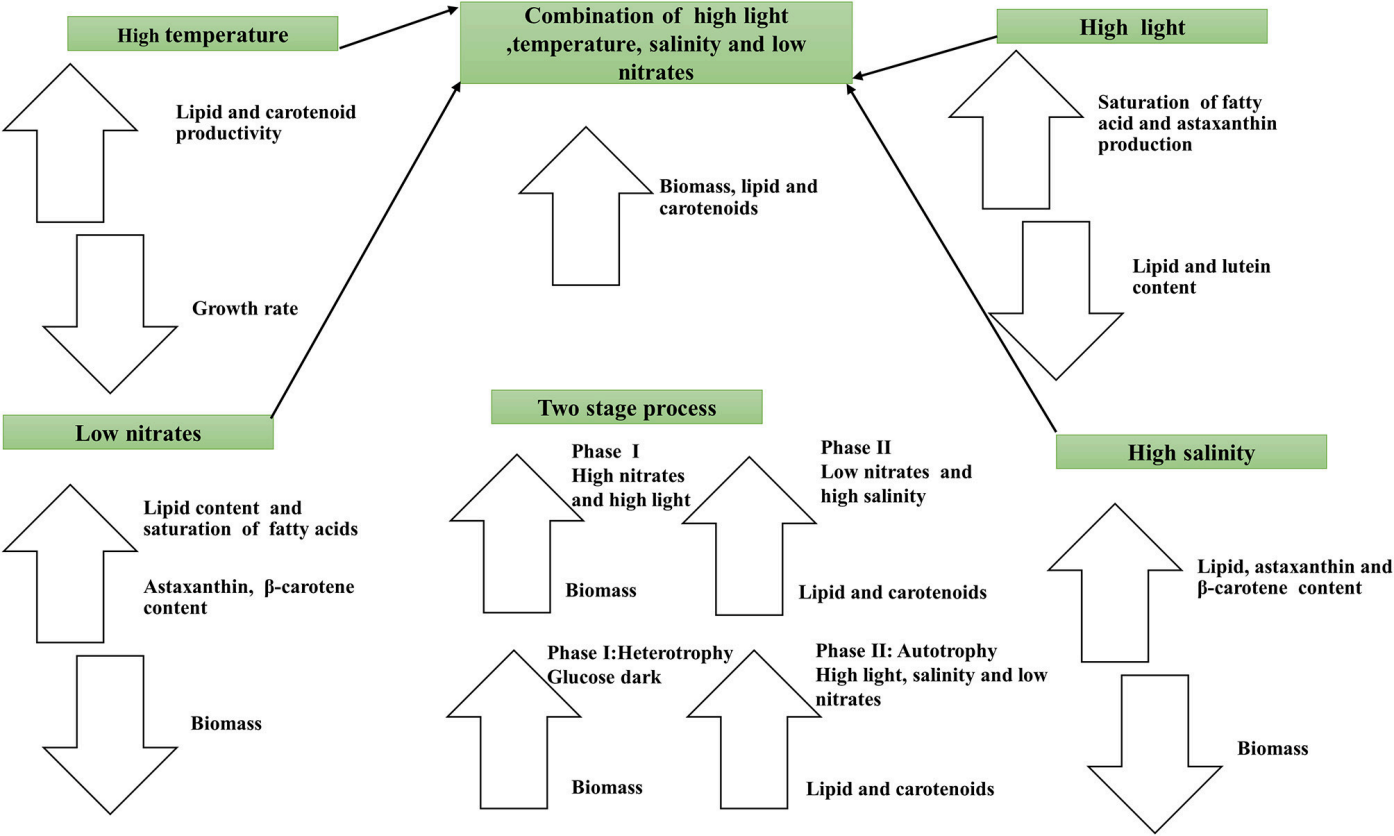
**Schematic diagram showing impact of environmental stresses on the lipid and carotenoids production**.

Autotrophic and heterotrophic strategies have been studied widely, and several physicochemical stress factors that affect the metabolism of microalgae significantly have been identified. Managing environmental stress is a typical approach used in refining microalgae based lipid production in the laboratory and at the pilot-scale. The most common stress factors used for enhancing lipid production are light intensity, temperature, and nitrates (Figure 2).

### Autotrophic growth factors

#### Temperature

Temperature is a stress factor that greatly influences the rate of growth, net lipid productivity and FA profiles in a wide range of microalgal species (Ho et al., 2014a). As microalgal growth and secondary metabolic pathway vary with temperature (James et al., 2013), greater knowledge of the biochemical response to temperature may yield useful insights into developing efficient systems for biofuel production (Wei and Huang, 2015). Normally, higher growth rate of microalgae is achieved by increasing the temperature to its optimum level (González-Fernández et al., 2012).

Studies on a large number of species have shown that both low and high temperatures can boost lipid productivity (Converti et al., 2009; Xin et al., 2011). Net lipid productivity decreased as temperatures increased from 25 to 35°C in *Monoraphidium* sp. SB2 (Wu et al., 2013); from 15 to 20°C in *Nannochloropsis oculata* (Converti et al., 2009); LX1, from 20 to 30°C in *Scenedesmus* sp. (Xin et al., 2011); and, from 14 to 30°C in *Nannochloropsis* sp. (Hu et al., 2006; Table 1). Both low and high temperatures are preferred for attaining higher lipid profiles, depending on the species: the levels of unsaturation in FAs increase under low temperatures, whereas those of total saturated FAs increase at high temperatures, as observed by Liu et al. (2005). The level of unsaturation is high at low temperatures mainly because of the higher concentration of dissolved oxygen (DO), which allows the oxygen-dependent enzymes, known as omega-3 desaturases, to function (Ward and Singh, 2005).

**Table 1:** **Effect of stress factors on lipid and bioactive production**.

**Micro-algal species**	**Operation strategy**	**Net lipid product-ivity (g/L)**	**Astaxanthin**	**Notes**	**References**
*Chlorella vulgaris*	High light intensity 2664–9324	27.0			Gonçalves et al., 2013
	25°C; N, 0.15 g/L	14.7		Omega-3, 65%	Converti et al., 2009
	N, 0.61–2.5 g/L	5.5–3.5			Prîbyl et al., 2012
	Glucose (20.0 g/L)	38.0			Kong et al., 2011
*Nannochloropsis* sp.	14–30°C	7.2–4.7			Hu et al., 2006
*Nannochloropsis oculata*	15–20°C	14.9–7.9			Converti et al., 2009
*Scenedesmus* sp.	20–30°C	11.2–7.4			Xin et al., 2011
*Monoraphidium* sp.	25–35°C	18.7–14.7			Wu et al., 2013
*Scenedesmus* sp.	Light intensity 50–250 μmol/m^2^s;	0.7–1.60			Liu et al., 2012
	NaCl,High light and 0.13 g/L NaNO_3_	59.0 mg/L/d	0.59 mg/g	Oleic acid 37.1%	Peng et al., 2012
*Mychonastes homosphaera*	Light intensity 7400 −29 600 lux	17.4–31.2			Tang et al., 2011
*Raphidocelis subcapitata*	Light intensity 2664–9324	9.0–39.0			Gonçalves et al., 2013
*B. braunii*	High light intensity	46.8			Ruangsomboon, 2012
	18°C	37.1			Kalacheva et al., 2002
	18°C	38.3			Sushchik et al., 2003
	0.1% NaCl; BG11 medium			Lutein 84.3%	Ranga Rao et al., 2010
	Salinity: NaCl + sodium acetate		15.14%	Lutein 68.4%	Ranga Rao et al., 2010
*Scenedesmus* sp.	30°C	7.4			Xin et al., 2011
	N, 0.002 –0.025 g/L	5.1–16.2			Xin et al., 2010
	N, 1.5 g/L	24.0			Rodolfi et al., 2009
*Auxenochlorella protothecoides*	N, 2.4–6.0 g/L	5.9–4.7			Shen et al., 2009
	Urea 1.0 g/L	9.1			Kong et al., 2011
	Glucose, 30.0 g/L	234.0			Heredia-Arroyo et al., 2010
	Crude glycerol	14.6			Chen et al., 2011
	35°C			Lutein 4.6%	Shi et al., 2006
	20 g/L NaCl	43.4%			Campenni et al., 2013
*Scenedesmus almeriensis*	High temperature			Lutein 4.5%	Del Campo et al., 2007
	30°C			Lutein 0.46%	Sánchez et al., 2008
*Muriellopsis* sp.	High temperature			Lutein 4.3%	Del Campo et al., 2007
*Haematococcus pluvialis*	Addition of Fe^2+^-EDTA		3.1%		Cai et al., 2009
	High light		9.5 μg/mL	Total fatty acid 133.2 μg/mL	Zhekisheva et al., 2005
	N deficient + light		3.0%		Imamoglu et al., 2009
	N deficient + light		21.8 mg/g		Orosa et al., 2000
	Heterotrophy		0.5%		Kobayashi et al., 1992
	High light intensity and mixotrophic conditions		10%		Domínguez-Bocanegra et al., 2004
	High temperature		5.5%		Chekanov et al., 2014
	N, 1.5 g/L	–	65.0 pg/cell		Boussiba and Vonshak, 1991
	0.8% NaCl	–	80.0 pg/cell	Carbohydrate 48%	Boussiba and Vonshak, 1991
	28°C; 25 530 lux	–	98 mg/g		García-Malea et al., 2009
	Phosphate starvation	43%			Saha et al., 2013
*Scenedesmus obliquus*	0–50 mM NaCl	18–28%		Oleic acid 28.3–41.1%	Salama et al., 2013
*Nitzschia laevis*	Urea+ Nitrogen			EPA 175 mg/L/d	Wen and Chen, 2001
*Nannochloropsis* sp.	14°C	7.2		PUFA 11.7%, EPA 7.9%	Hu et al., 2006
	22°C			EPA 25.3%	Hu et al., 2006
	15°C	14.9			Converti et al., 2009
	N, 1.5 g/L	13.5			Rodolfi et al., 2009
	N, 0.012 g/L	13.6			Hu et al., 2006
	High CO_2_	9%			Hu and Gao, 2003
*Chromochloris zofingiensis*	24–28°C	–	12.3 mg/L	Lutein 21 mg/L	Del Campo et al., 2004
	N, 2.5 g/L	–	18.0 mg/L	Lutein 25 mg/L	Del Campo et al., 2004
	High light intensity	–	19.0 mg/L	Lutein 24.7 mg/L	Del Campo et al., 2004
	Zero salinity + light	–	15.0 mg/L	Lutein 15.0 mg/L	Del Campo et al., 2004
	Glucose, 50.0 g/L	–	10.0 mg/L		Sun et al., 2008
	Glucose, 50.0 g/L	–	10.2 mg/L		Feng et al., 2005
	300 mM NaCl		0.8 mg/L		Orosa et al., 2001
*Chlorella zofingiensis*	N deficiency	65.1%			Feng et al., 2012
	P deficiency	47.7%			Feng et al., 2012
	High light intensity		1.5%		Del Campo et al., 2004
	0.2 M NaCl		4%		Del Campo et al., 2004
	Mixotrophy		12.5 mg/L		Ip et al., 2004
	Heterotrophy		32.0 mg/L		Ip et al., 2004
*Dunaliella salina*	High light intensity	–		β-carotene 3.1%	Lamers et al., 2010
	N, 2.5 mM 16% NaCl	37.7%			El-Baky et al., 2004
	N starvation			β-carotene 2.7%	Lamers et al., 2012
*Dunaliella tertiolecta*	N, 0.026 g/L; NH_4_Cl (0.16 m)			PUFA 73%	Chen et al., 2011
*C. vulgaris* AG10032	Two-stage nitrogen depletion	77.1 mg/L/d			Mujtaba et al., 2012
*N. gaditana*	Two-stage (continuous culture + nitrate depletion)	51.0 mg/L/d			San Pedro et al., 2013
*Auxenochlorella pyrenoidosa*	Semi-continuous (low-N medium replacement)	115.0 mg/L/d			Han et al., 2013
	N, 0–0.4 g/L	1.4–6.66 g/L			Singh et al., 2011
*Chlamydomonas* sp. JSC4	Salinity: NaCl	223.2 mg/L/d			Ho et al., 2014b
*Arthrospira* (Spirulina) *platensis*	Phosphate limitation			Carbohydrate 67%	Markou et al., 2012

Besides lipids, ratio of saturated FAs to unsaturated FAs is species dependent but only partly because of innate differences in the ability of the species to synthesize lipids (either DHA or other FAs) under low temperatures. In species such as *Nannochloropsis* sp. and *Phaeodactylum tricornutum*, high (31.7 and 24.2%, respectively) content of EPA C20:5 (omega-3) have been reported at low temperature stress (Jiang and Gao, 2004; Hu et al., 2006). Further, temperature is also known to affect carbohydrate production in microalgae; for example, in *Spirulina* sp. carbohydrate content increased up to 50% when temperature was increased from 25 to 40°C (Ogbonda et al., 2007). It seems that in many microalgal species temperature changes from low to high increases the lipids productivity. High temperature also favor biofuel properties. We have also noted that temperature stress is strain dependent and all strains do not thrive equally at high temperature. Therefore, temperature is a crucial stress factor to be taken in to account for optimizing lipids and biomass productivity.

#### Light

Light can bring significant changes in the chemical composition of microalgae. It is essential for photosynthesis, and together with photoperiod is a critical factor for microalgal growth (Wahidin et al., 2013). Light intensities of 100–200 μE/m^2^/s are commonly used for microalgal production (Zhao and Su, 2014). For example, increasing the light intensity from 200 to 400 μE/m^2^/s increased the rate of growth in microalgae (Radakovits et al., 2010). In *Mychonastes homosphaera, Chlorella vulgaris, Raphidocelis subcapitata*, and *Scenedesmus* sp. (Tang et al., 2011; Gonçalves et al., 2013; Liu et al., 2012), lipid productivity increased with increase in light intensity from low to high as shown in Table 1. Khotimchenko and Yakovleva (2005) reported high content of PUFA under low light intensity whereas high light intensity resulted in greater accumulation of saturated FAs and monounsaturated FAs.

Optimum light requirements varies with the microalgal species, and several parameters need to be considered in selecting the right combination when using artificial light in view of the overall energy balance considerations. It may be feasible to use high light intensities for enhanced production of lipids, biomass as well as suitable fatty acid profile for improving biofuel potential. Lipids productivity is therefore found to be influenced under high light stress. A serious concern is that the cells experience photoinhibition at high light. Thus, cultivation of microalgal strains in a two phase system, where in first phase cells are cultivated under low light and then shifted to high light in second phase can overcome the limitation caused by high light alone. This strategy may improve the overall biomass and lipid productivity and addresses appropriately the concern of photoinhibition.

#### Nitrate

Of all the macronutrients in the culture medium, nitrogen, which accounts for 1–10% of the total dry matter in microalgae (Wijffels et al., 2010), is quantitatively the most important nutrient affecting growth and lipid accumulation in various algae (Griffiths and Harrison, 2009). Nitrogen in different forms such as ammonium, nitrate, yeast, peptones, and urea, when used as a nutrient supplement, changes the rate of cell metabolism significantly (Chen and Chen, 2006) and plays a significant role in lipid synthesis (Figure 1). Increased lipid accumulation under nitrogen starvation conditions in several microalgae is well documented. For example, under nitrogen starvation, lipid production increased about twofold and onefold in *Neochloris oleoabundans* and *Nannochloropsis* sp. F&M-M24 (Li et al., 2008; Rodolfi et al., 2009), respectively. Nitrogen deficiency also affects the biosynthetic pathway of carbohydrates (Cheng and He, 2014). For example, carbohydrate content increased fourfold in *Tetraselmis subcordiformis* and by 29% in *Scenedesmus obliquus* CNW-N under nitrogen deficient conditions (Ji et al., 2011; Ho et al., 2012). Restricted supply of nitrogen and phosphorus shifts lipid synthesis from membrane lipids to neutral lipids (Juneja et al., 2013).

Moreover, lipid productivity increased with increasing nitrate concentration in *Auxenochlorella pyrenoidosa* (Singh et al., 2011) and *Scenedesmus* sp. (Xin et al., 2010), but the exact opposite pattern was observed in *Auxenochlorella protothecoides* (Shen et al., 2009) and *C. vulgaris* (Prîbyl et al., 2012), in which lipid productivity was higher at lower concentrations of nitrate (Table 1). A serious concern about cultivating microalgae under nitrogen limiting condition lowers biomass and consequently decrease the overall lipid productivity.

Two nitrogen limitation strategies has been defined to boost the lipids production in several microalgae. The first one is two stage nitrogen depletion as shown in *C. vulgaris* AG10032 (Mujtaba et al., 2012) in which microalgal cells are grown in nitrogen rich conditions to stimulate biomass growth and then transferred in to nitrogen starved medium for lipids production. The second one is one stage cultivation in which the medium contains desired nitrogen concentration and then as culture grows the nutrient starts depleting and finally starved conditions is achieved (Yen et al., 2013). The lipid and biomass accumulation in both of these strategies is species-dependent. Additionally, when nitrogen is deficient or lacking, many cyanobacteria produce hydrogen as a result of nitrogen fixation (Abed et al., 2009).

Thus, nitrate levels must be controlled throughout the growth in culture to maintain the nutrient contents of a medium stable and thereby prevent damage to the cell membrane, loss of biomass, and other changes in cell composition. Thus, screening based on lipid productivity, biomass production, and FAME profiles among strains grown under common parameters can achieve the highest resource utilization efficiency and maintain a positive material and energy balance under low cost nitrogen starvation technique. It is clear that under nitrogen limitation, the lipid production is increased in all microalgal species. However, biomass limitation under nitrogen deficient conditions is major concern, as lower biomass tends to reduce the net lipid productivity. Thus, cultivating microalgae in two phase system as described above can overcome the limitation of biomass production.

#### Phosphate

Phosphorous makes up considerably less than 1% of total algal biomass (~0.03–0.06%) and yet is an essential component in the medium for sustainable growth and development of microalgae (Hu, 2004; Hannon et al., 2010). Absence of phosphorus in a medium results in repression of photosynthesis (Belotti et al., 2014) and affects the growth of microalgae. However, phosphorus has a significant impact on the growth and metabolism of microalgae (Fan et al., 2014).

It has been observed that limitation of phosphates resulted in increased lipid accumulation in microalgal cells. For example, in the freshwater microalgae *Scenedesmus* sp. LX1 (Xin et al., 2010) and *Botryococcus braunii* (Venkatesan et al., 2013), net lipid productivity increased under phosphorus-limited (stress) conditions. Another study, by Markou et al. (2012), showed that under phosphorus-limitation, carbohydrate content increased from 11 to 67% in *Arthrospira* (Spirulina) *platensis* (Table 1). Thus, while designing the strategies to achieve higher lipid and biomass under environmental stresses combination of low nitrates and optimal phosphate concentration may increase the lipid and biomass production in one growth cycle. Developing such strategies in future can make the process more cost effective.

#### Ammonium

Ammonium is considered an energy-efficient source of nitrogen for microalgae because its uptake requires less energy (Li X. et al., 2010). In *Cylindrotheca fusiformis* and *P. tricornutum*, transport of ammonium may not be up-regulated by limiting nitrogen; however, when these species are transferred to a nitrate-rich medium, the ammonium transporters are expressed (Hildebrand, 2005). Ammonium as a substrate influences namely, PUFA content in *Dunaliella tertiolecta* (Table 1) but its influence on net lipid productivity is unknown and needs to be studied in a more diverse range of species. Although ammonium uptake by cells can lower the growth of microalgae, however is still a preferred nitrogen source in many cases as conditions can be controlled during microalgae cultivation to minimize any negative impact on growth.

#### Salinity

Salinity is an intricate stress factor affecting net lipid productivity in microalgal cells. Microalgal species can tolerate salinity stress up to an extent. Effects of salinity on marine algae have been examined by several researchers (Bartley et al., 2013; Martinez-Roldan et al., 2014) however limited reports are available on freshwater microalgae. *Dunaliella* sp. is one such species that can tolerate high salinity stress and produce large quantities of lipid and biomass (Azachi et al., 2002). For example, in *D. tertiolecta* ATCC 30929 under high salinity concentration, increased lipid content up to 70% (Takagi et al., 2006). In the freshwater alga *Scenedesmus* sp., NaCl is believed to stimulate higher lipid production (Salama et al., 2013). However, excess salinity stress in cultivation medium inhibits photosynthesis which further reduces the biomass and net lipid productivity.

As discussed above, salinity stress tends to remarkably affects the fatty acid profile of microalgae. The changes in each fatty acid chain is species specific. Thus, cultivating microalgae in combination with salinity and nitrate stress in lab scale can further enhance the production of lipids and cell biomass in a single life cycle. A correlation between changes in the production of lipids and photosynthetic efficiency has not been well-studied across diverse microalgal strains.

Since NaCl is an inexpensive and readily available source of nutrients, adjusting the concentration of NaCl along with light intensities could further enhance the production of lipid, biomass, and biofuel properties in diverse microalgal species and hence can lower the cost of biofuel production. The effects of NaCl in combination with those of other stress factors therefore needs to be examined further.

#### Carbon

Various concentrations and sources of carbon affect the overall composition of microalgal lipids. Both low and high concentrations of carbon dioxide have shown to induce the accumulation of saturated and unsaturated FAs, respectively (Hu and Gao, 2003). Besides carbon dioxide, several microalgae use other carbon sources for production of lipids and other compounds under heterotrophic conditions, as described in the section on “Heterotrophic growth factors.”

### Heterotrophic growth factors

Heterotrophic organisms are those that lack the photosynthetic machinery and cannot generate energy through oxidation of inorganic compounds and therefore need an external carbon source. Carbon sources such as glucose, acetate, and glycerol are the most important element for the production of lipids and other compounds. The research on heterotrophic cultivation remained focused on *Chlorella* sp.

#### Glucose

Glucose is most preferred by diverse microalgal species as it can be easily assimilated and produces energy-rich compounds such as neutral lipids as storage products. Microalgae grown with glucose have shown higher growth rates than those grown on acetate and fructose as it produces more energy (~2.8 kJ/mol) in comparison to acetate (0.8 kJ/mol) and stimulates the metabolic pathways involved in starch and lipid synthesis (Boyle and Morgan, 2009). Two major factors that affect the growth of heterotrophic microalgae are the innate ability of a strain and the conditions under which it is cultured. However, the overall consumption of carbon is ultimately determined by how efficiently it is metabolized and transported through the cell membrane (Azma et al., 2011). Many reports (Heredia-Arroyo et al., 2010; Kong et al., 2011, for example) show that a carbon source such as glucose at 30 g/L and 20 g/L increased the net lipid productivity in *A. protothecoides* and *C.vulgaris* (Table 1). *Chlorella* sp. grown heterotrophically produced 260% more lipids and 45% more total carbohydrates than that grown autotrophically (Miao and Wu, 2006). Similarly *C. protothecoides* grown heterotrophically also showed fourfold higher lipid than that grown autotrophically (Xu et al., 2006). In almost all freshwater algae including *Chlorella* sp. (Leesing et al., 2011), *N. oculata* (Wan et al., 2011), and *Chlorella sorokiniana* (Wan et al., 2012), net lipid productivity increases with increase in glucose concentration as long as light is not a limiting factor. In *C. sorokiniana*, metabolic glucose flux occurs through glucose-6-phosphate dehydrogenase in which glucose-6-phosphate isomerases act as a catalyst, and this species is known to generate more energy in the form of ATP than that generated by the autotrophic strain with light as energy source (Yang et al., 2000). It is evident that in *Chlorella*, the transport of ion across the cell membrane is proton dependent. *Chlorella* cells have an effective hexose/H^+^ transport system through which glucose from the medium is utilized (De Swaaf et al., 2003). The metabolism of glucose is different and complex under autotrophic and heterotrophic conditions, and the pathways are also quite different.

Under heterotrophic conditions, glucose is utilized in the cell by means of the pentose phosphate pathway (Figure 1) whereas under autotrophic conditions, the Embden–Meyerhof pathway becomes functional (Yang et al., 2000). Efforts have been made to increase the lipid production by controlling metabolic growth during cultivation. Two stage cultivation process where in first heterotrophic cultivation (glucose) can obtain higher biomass and then shift to autotrophic mode (light) to achieve the desired products could influences net lipid productivity positively (Zhang et al., 2014). Although it is generally agreed that glucose is most preffered source of carbon. The effect of glucose on metabolism of microalgae varies significantly. Cultivation of microalgae in medium amended with glucose in presence of light can improve the biomass and lipids production in diverse microalgal species. In summary, information on concentration of glucose to be used for metabolic growth of algae is too diverse to reach some specific conclusions.

#### Glycerol

Glycerol is another carbon source that provides energy under heterotrophic conditions (Perez-Garcia et al., 2011). Glycerol boosts lipid productivity in freshwater microalgae but no data are available on its effect on the FA profile. Glycerol alone or in combination with glucose affects net lipid productivity: as shown in *A. protothecoides* (Heredia-Arroyo et al., 2010) and *C. vulgaris* (Kong et al., 2011). At higher concentration stress increases lipid productivity under mixotrophic conditions with low concentrations of glucose.

Organic carbon assimilation under mixotrophic conditions induced changes in both respiratory and photosynthetic metabolism in cyanobacteria (Vonshak et al., 2000); however, supplementing the organic carbon through glucose and that of energy in the form of light may convert the metabolic pathway in microalgal cells. Biochemical composition is thus influenced by light and by the concentration of nutrients in the medium (Kong et al., 2013). For example in *P. tricornutum* cultivated with 0.1 M glycerol at 165 mmol photons/m^2^/s light intensity, growth increased up to 74% compared to autotrophic culture (Cerón et al., 2007). On the other hand, *A.* (*Chlorella*) *protothecoides* cultures with glycerol produced only half yield of total lipids that of cultures grown with glucose (Cerón-García et al., 2013). Grown on crude glycerol *A.* (*Chlorella*) *protothecoides* produced similar lipid and biomass to that grown in presence of glucose (Chen et al., 2011).

#### Urea

Urea is another carbon source that subsequently influences cell metabolic activity significantly. It affects the growth of microalgae, although very few studies have shown how it affects lipid productivity within the cell (Neilson and Larsson, 1980). Urease and urea amidoylases are the enzymes which help in metabolizing urea. However, it is unfortunate that *Chlorella* strains lack the enzyme urease (Kaplan et al., 1986), and the metabolism of urea is accompanied by ualse. However, some *Chlorella* sp. uses urea as a source of nitrogen (Oh-Hama and Miyachi, 1992; Table 1). Thus, allowing the enzyme allophanate lyase in the ualse pathway helps in hydrolysing and converting urea to ammonium and bicarbonates (Morris, 1974).

Strategies using a combination of urea and nitrogen can promote the production of EPA in *Nitzschia laevis* (Wen and Chen, 2001). The more indepth research efforts are needed to understand role of urea and its impact on multidirectional metabolic pathways. Cultivation of microalgae under high urea concentration can increase the biomass production in different microalgae. All microalgae strains cannot assimilate urea therefore selection of strain and their cultivating conditions needs major attention.

## Microalgal synthesis of carotenoids

Microalgae are known to produce pigments and bioactive compounds, of which three are particularly promising: chlorophyll, phycobilins, and carotenoids (Abalde et al., 1991). Chlorophyll is mainly found in algae, cyanobacteria and higher plants. Carotenoids are divided in to primary and secondary carotenoids. Lutein is considered a major carotenoid since it acts as a primary carotenoid maintaining the membrane integrity of cells and protecting cells from many forms of stress (Sánchez et al., 2008), whereas astaxanthin is considered a secondary carotenoid, present in lipid bodies outside the chloroplast (Grünewald et al., 2001), and has potential applications in human health. Astaxanthin and canthaxanthin are considered major ketocarotenoids in algae, fungi, and bacteria (Abe et al., 2007) and therefore of great interest for biotechnological applications and as feed supplements in aquaculture (Yaakob et al., 2014).

In plants and algae, carotenoids are synthesized in the chloroplasts around the nucleus and their accumulation is effected only when they are exposed to some stress factors (Lemoine and Schoefs, 2010). Astaxanthin accumulation in lipid bodies outside the chloroplast increases under several stress conditions (Wang and Peng, 2008). Phycobiliproteins are water soluble pigments found in cyanobacteria and eukaryotic algae (MacColl, 1998). The above pigments are high-value compounds, produced from *Porphyridium cruentum, Synechococcus* sp., and *Chlorella* sp. (Rodrigues et al., 2014). Due to their color, carotenoids are used in dyes and as coloring agents (Yuan et al., 2011; Christaki et al., 2013).

Astaxanthin, β-carotene, and lutein are the main promising microalgal pigments with high market potential (Table 2). The above pigments are vital for the survival of a cell because they form the basic and functional components of photosynthesis in the thylakoid membrane (Guedes et al., 2011). Other pigments such as xanthophylls, mostly found in cyanobacteria, are well linked with chlorophyll-binding polypeptides (Grossman et al., 1995); however, in a few green microalgae, xanthophylls are localized, and synthesized within the plastid, whereas in *Haematococcus* sp., astaxanthin accumulates in the cytoplasm (Eonseon et al., 2003). Carotenogenesis pathways and relative enzymes have been studied in cyanobacteria (Takaichi and Mochimaru, 2007). The enzymes and genes involved in carotenoid synthesis are different in algae and cyanobacteria (Takaichi, 2011). *Haematococcus pluvialis, D. salina*, and *Chlorella* sp. are well-studied candidates for the production of commercially important carotenoids (Gimpel et al., 2015) (Table 2). Furthermore, in photosynthetic organisms these carotenoids play important roles in either absorbing energy or protecting cells from photo-oxidative damage (Demming-Adams and Adams, 2002). Although, the pigments are basically associated with exposure and duration of light intensity. The production of metabolites under dark conditions is yet to explored.

**Table 2:** **Market potential of products**.

**Bioactive compound**	**Mode of production**	**Cultivation method**	**Applications**	**Key players**	**Market (USD, million)**
Nutraceutical astaxanthin	*Haematococcus pluvialis*, freshwater green alga (Chlorophyceae)	Tubular phorobioreactor (Two phase)	Natural anti-oxidant; prevents age- related macular degeneration, neuro-degenerative diseases, dyspepsia, sunburn, hypertension, benign prostrate hyperplasia; improves sperm fertility, muscle function, normalizes cardiac rhythm; helps in stress management and stroke repairs	AlgaTech, Israel (a)	200
				Blue Biotech, Germany (b)	
				Fuji Chemicals, Japan (c)	
				BioReal, Sweden (d)	
Biorefinery carotenoids (astaxanthin, lutein), PUFA, lipids and proteins	Chromochloris zofingiensis, freshwater green alga (Trebouxiophyceae)	Tubular photobioreac-tor one stage	Defends cells from damaging effects from free radicals and age-related macular degeneration and cataract	Blue Biotech, Germany (b)	40
				Earthrise, USA and Dainippon, Japan (e)	
				Chlorella Co., Taiwan (f)	
EPA	*Phaeodactylum tricornutum*, hetrokont, diatoms	Hybrid raceway	Produced for cosmetics and aquaculture; anti-inflammation (peptide) used as feed additives	Soliance (g)	300
	Nannochloropsis spp. Heterokonts (Eustigmatophyceae)	Hybrid two- stage		Blue Biotech, Germany (b)	
DHA	Schizochrytium, Cryptocodinium		Food supplement (omega-3 fatty acid, brain development for children)	Xiamen Huison Biotech Co. (China), Martek (USA)	1.5 billion
Fucoxanthin	Laminalia japonica (Laminariaceae)		Prevents liver and skin cancer owing to its ant-ioxidant activity, and breast and prostate cancer through induction of apoptosis	AlgaNova International, China	Yet to reach 100
Phycocyanin	Spirulina platensis (Phormidiaceae)	Open ponds, Natural lakes	Used in immunofluorescent labeling; as natural colors in food and cosmetics, anti-oxidant, anti-tumor, anti-cancer agents; prevents atherosclerosis; exerts inhibitory effects, induces allergic inflammatory response, and helps in stem cell regeneration especially in bone marrow and blood cells	Parry nutraceuticals, SandaKing (Japan) (Photonz. Corporate)	50
β-carotene	Dunaliella Salina	Open raceway	Natural colorant; prevents skin, ovarian and breast cancers, solar keratosis, arthritis, bronchitis	Nutrition & Health, Australia; Cyanotech, Hawaii, USA (h)	261

(a)www.algatech.com (2013).

(b)www.bluebiotech.de/(2013).

(c)www.fujichemical.co.jp (2013).

(d)www.bioreal.se (2013).

(e)www.earthrise.com (2013).

(f)Chacon-Lee and Gonzalez-Marino (2010).

(g)www.soliance.com/ (2013).

(h)http://www.reportlinker.com/p096628-summary/The-Global-Market-for-Carotenoids.html Last accessed 29/6/2013.

## Strategies to enhance microalgal carotenoids production

Secondary carotenoids synthesis is altered by changes in culture conditions and their production can be enhanced by controlling several stress factors (Lemoine and Schoefs, 2010) as shown in schematic Figure 2. Carotenogenesis is enhanced by reactive oxygen species (ROS), under stress conditions such as high light intensity, salt stress (Kobayashi et al., 1993). Because of this, astaxanthin is believed to protect the body from such free-radical-linked diseases as oral, colon, and liver cancers (Guerin et al., 2003).

Several unfavorable environmental conditions such as nutrient deficiency, intense irradiation, and excessive photosynthesis lower the rate of electron transfer and, in turn, photo-oxidative damage (Solovchenko et al., 2011). Primary carotenoids, such as lutein degrade under stress and therefore their biomass content is decreased. However, several primary carotenoids, such as β-carotene act as secondary metabolite and therefore accumulate under stress conditions (Rabbani et al., 1998). Carotenoids content and accumulation in high concentration in total biomass varies under several stress factors. Combined effect of several stress factors have enhanced the production of astaxanthin in *H. pluvialis* (Ben-Amotz, 2004).

Two stage cultivation process (Aflalo et al., 2007), continuous process (Zhang et al., 2009) have been opted by researchers for increasing the astaxanthin production in microalgae. Under stress conditions, β-carotene is accumulated in lipid bodies with in the chloroplast with highest content of 12% dry cell weight (Del Campo et al., 2007). Similar to astaxanthin, β-carotene content in microalgal species increases when growth is arrested under several stress factors (Lamers et al., 2012). Lutein production in *Muriellopsis* sp. and *Scenedesmus almeriensis* is unaffected under stress conditions (Table 1) unlike astaxanthin and β-carotene (Del Campo et al., 2000). However, these two species have been tested in large scale cultivation system for commercial purpose (Fernández-Sevilla et al., 2010). Autotrophic and mixotrophic cultivation has been studied in detailed below to develop the method for enhanced production of metabolites.

### Autotrophic growth factors

#### Temperature

Temperature plays an important role in the accumulation of carotenoids within microalgal cells. Higher temperatures result in increased accumulation of carotenoids in microalgal species due to increased photo-oxidative stress (Tripathi et al., 2002). Temperature affects the synthesis of astaxanthin in *H. pluvialis* (Chekanov et al., 2014) and *Chromochloris zofingiensis* (Del Campo et al., 2004) as depicted in Table 1. High temperature is rarely used for inducing astaxanthin production because it is reported to reduce biomass yield drastically, thereby leading to an overall reduction in astaxanthin production.

Temperatures above 28°C were found to decrease the overall productivity of astaxanthin in C. *zofingiensis* (Del Campo et al., 2004) because ROS are assimilated in greater quantities during photosynthesis, which causes the accumulation of astaxanthin (Kovacic, 1986). In *Dunaliella salina*, high temperature results in accumulation of lutein (García-González et al., 2005). In addition, in *S. almeriensis* the lutein content and net biomass increased to 0.46% and 0.53 g/L, respectively, when temperatures reached up to 30°C (Sánchez et al., 2008). A similar trend was also observed in *C. protothecoides* (Shi et al., 2006) (Table 1). In contrast low temperature increased β-carotene and α-carotene content in *Dunaliella* sp. (Gómez and González, 2005). However, overall lutein content was affected when temperature was increased from 20 to 33°C in *Muriellopsis* and *S. almeriensis* (Del Campo et al., 2007) in one growth cycle (Table 1).

Temperature is the only factor which enhanced the production of lutein. Temperature effect on carotenoids production is not well-known, however it is observed high temperature tends to accumulate more lutein content where as it has no effect on astaxanthin and β-carotene production. Autotrophic cultivation of microalgae under temperature stress is species dependent. For enhanced production of carotenoids, cultivating microalgal strains at high temperature in combination with other stress factors such as light and salinity could be more effective.

#### Light

Light availability is one of the most serious limiting factors for production of several carotenoids, biomass (Cordero et al., 2011), and FAs (Ward and Singh, 2005). Little is known about carotenoids metabolism as affected by the quality of light (Fu et al., 2013). Light intensity as well as photoperiod affect growth, biomass, and other metabolites of interest in diverse microalgae species (Seyfabadi et al., 2011; Khoeyi et al., 2012). Increasing the light intensity resulted in a threefold increase in astaxanthin in (Table 1) *H. pluvialis* (Del Campo et al., 2004). Besides astaxanthin, β-carotene content was also increased in *D. salina* up to 3.1% of dry cell weight (Table 1) when light intensity was changed from 100 to 1000 μmol photons/m^2^/s (Lamers et al., 2010). Lutein content is also affected under high light intensity; for example, in *Muriellopsis* sp. lutein content peaked at 460 μmol photons/m^2^/s. High light intensity alone helps in promoting the growth of microalgae but reduced overall lutein content in *Scenedesmus* sp. (Xie et al., 2013). In all the above species, greater exposure to light increases the concentration of astaxanthin and lutein.

The production of lutein was studied in *C. sorokiniana* (Cordero et al., 2011) and *Scenedesmus* sp. (Sánchez et al., 2008) because both the species have a high growth rate and high lutein-accumulating power. On the other hand, formation of primary β-carotene and SC is affected by low as well as by high light intensity: although photosynthesis occurs under both levels, under low light intensity it generates fewer oxygen radicals, whereas under high light intensity, cells are unable to utilize all the energy that is generated, and the surplus energy activates more oxygen molecules, leading to the formation of SC such as astaxanthin (El-Baz et al., 2002). Such an exception has not been reported in *H. pluvialis*, in which cells continue to generate oxygen molecules during photosynthesis. In summary, carotenogenesis in photosynthetic cells is correlated with different developmental changes and depends upon environmental conditions. It is observed that, high light favors the production of astaxanthin and β-carotene. Cultivation of microalgae under high light stress in combination with nitrates can further enhance the production of biomass and carotenoids (like astaxanthin, lutein and β-carotene).

#### Nitrate

Nitrate as a stress factor influences not only lipid production but also affects carotenoids accumulation in green algae. Several factors trigger carotenoids synthesis and should be considered in developing microalgal products. Limiting nitrogen in the growth medium increases the production of SC but lowers overall biomass yield (Cordero et al., 2011). Ammonium nitrate have less impact on microalgal growth and carotenogeneis (Markou and Nerantzis, 2013). Moreover, total astaxanthin content of 3% dry cell weight (Table 1) was obtained in a nitrogen-deficient medium (Imamoglu et al., 2009). Absence or low levels of nutrients including nitrogen stimulate rapid physiological responses, which further trigger the secondary biosynthetic pathways (Touchette and Burkholder, 2000).

Lutein accumulation in microalgae cells is highly dependent upon nitrogen concentration within the medium (Del Campo et al., 2000). Under nitrogen-limiting conditions, carotenoids (β-carotene, astaxanthin, and canthaxanthin) begin to accumulate in aerial microalgae (e.g., *Coelastrella* sp. and many more), and the color of cells changes from green to red (Abe et al., 2007). *Chromochloris zofingiensis*, however, is an exception, and accumulates lutein and astaxanthin as the main compounds (Table 1). Besides lutein and astaxanthin, β-carotene content increased up to 2.7% of dry cell weight in *D. salina* (Lamers et al., 2012) under nitrogen-depleted conditions (Table 1). In general, nitrogen deficiency has a greater impact than excess nitrogen on carotenoids production, mainly that of astaxanthin in *H. pluvialis* (Table 1), because a culture growing in a nitrogen-rich medium requires carbon to assimilate the nitrogen. Low nitrogen, on the other hand, leads to greater competition for carbon, which is required for the synthesis of both carotenoids and proteins (Borowitzka et al., 1991).

Hence a combination of urea and other nitrogen sources achieved maximal lutein production in *A. protothecoides* under heterotrophic conditions (Shi et al., 2000). A two phase system where both nitrogen enriched and deficient medium is used in presence of high light intensity stimulate the growth of microalgae during first phase with biomass enhancement and in second phase deficient medium enhances carotenoids enrichment. These observations could be vital considerations toward defining future cultivation strategies.

#### Salinity

Salinity has diverse effects on species and varies greatly across habitats (Table 1). The effect of salinity on growth and carotenoids formation is complex. Several species of microalgae can tolerate a range of salinity levels because of efficient osmoregulation, which involves continuous synthesis of glycerol (Pick, 2002). For example, carotenoids production differs among the four species of *Dunaliella*, namely *D. salina, D. bardawil* (Gomez et al., 2003), *D. tertiolecta*, and *D. viridis* (Hadi et al., 2008): *D. tertiolecta* produces greater quantities of carotenoids at optimum salinity levels whereas *D. viridis* does so only at higher salinity levels. However, salinity affects overall β-carotene content (Coesel et al., 2008). Salt stress of 5.5 M increased carbohydrate content in *D. salina* (a 2.5-fold increase) (Mishra et al., 2008). Increase in salinity increases nitrogen and carbon content, which forces the cell to produce glycerol and amino acids to cope with the high salinity (Jiménez and Niell, 1991).

In *Chromochloris zofingiensis*, lutein and astaxanthin accumulate only during later stages of growth because both are synthesized from a common precursor, lycopene, with primary carotenoids as derivatives (Hirschberg et al., 1997). The astaxanthin content increased under salinity stress in *H.pluvialis* and C. *zofingiensis* (Table 1). However, *H. pluvialis* cannot tolerate salt concentrations above 10% w/v (Borowitzka et al., 1991), whereas concentrations higher than 2 mM of NaCl decreased total lutein content (Cordero et al., 2011) in *C. sorokiniana*. Salinity stress can further stimulate the production of total carotenoids. However, anything in excess causes harm to the metabolic system of microorganism. Thus, we inferred that NaCl stress have positive impact on increasing the astaxanthin and β-carotene production however it has no impact on the lutein production. Cultivating microalgal strains in the medium containing high nitrates and high salinity together can increase the production of lutein, astaxanthin and β-carotene, while using a two stage cultivation process. Combination of several environmental stresses in one culture medium could become a novel strategy for enhancing the metabolites production during single growth cycle. However, the metabolic pathway functioning of different stress factors in diverse microalgal strains is a complex phenomenon and has not been well-explored and analyzed.

#### Iron

Iron is needed for the growth of microalga such as *Dunaliella.* Of all the micronutrients, iron was the best for accumulation of astaxanthin in cysts of *H. pluvialis* (Kobayashi et al., 1993) because iron acts as a chelating agent; can scavenge hydroxyl radicals in the Fenton reaction widely used in the enzyme system of animals, microbes, and plants (Raven et al., 1999); and acts as limiting factor under hypersaline conditions.The site for assimilation of iron is usually the plasma membrane (Polle and Song, 2009). He et al. (2007) reported that phosphorus, iron, and sulfur are important nutrients for enhancing astaxanthin accumulation. However, supplementing the medium with Fe^2+^-EDTA (Table 1) produced biomass that contained 3.1% dry cell weight astaxanthin in *H.pluvialis* (Cai et al., 2009).

### Heterotrophic and mixotrophic growth modes

Published work on the effect of glucose on astaxanthin production is scant because microalgae cannot use external carbon sources efficiently. However, the ability to use external carbon varies with the species and culture conditions. Under heterotrophic conditions, glucose is used as a carbon source for up-regulation of some genes, namely *bkt, chy-b*, and *pds*, in *H. pluvialis, Chromochloris zofingiensis* (Sun et al., 2008), and *Synechocystis* sp. (Ryu et al., 2004). Glucose is reported to be the end product of photosynthesis, and any organism growing under light may be able to use glucose effectively (García-González et al., 2005). In *C. zofingiensis* (Sun et al., 2008) and *A. protothecoides* (Shi et al., 1997), the yield of astaxanthin increases with increase in glucose concentration. Whereas increase in glucose concentration further increases lutein content in *Chlorella* (Shi et al., 2000). Under heterotrophic conditions, *H. pluvialis* grows very slowly; however, it accumulates a total of 0.5% (dry cell weight) of astaxanthin (Kobayashi et al., 1992) as depicted in Table 1. Under mixotrophic conditions, both growth and astaxanthin production are increased (Wang et al., 2004). However, another study reported astaxanthin yield of 12.5 mg/L in *C. zofingiensis* under mixotrophic conditions (Ip et al., 2004), and in glucose-supplemented medium the yield increased to 32 mg/L (Table 1). Hence, different nutrients such as nitrogen and carbon affect growth and other parameters such as growth rate and pigment content, thereby affecting the biochemical composition of microalgae (Orosa et al., 2000). In summary, mixotrophic cultivation are more preffered for enhancing the biomass, lipid and carotenoids yield in microalgal species.

## High-value products from microalgae

**Figure 3:**
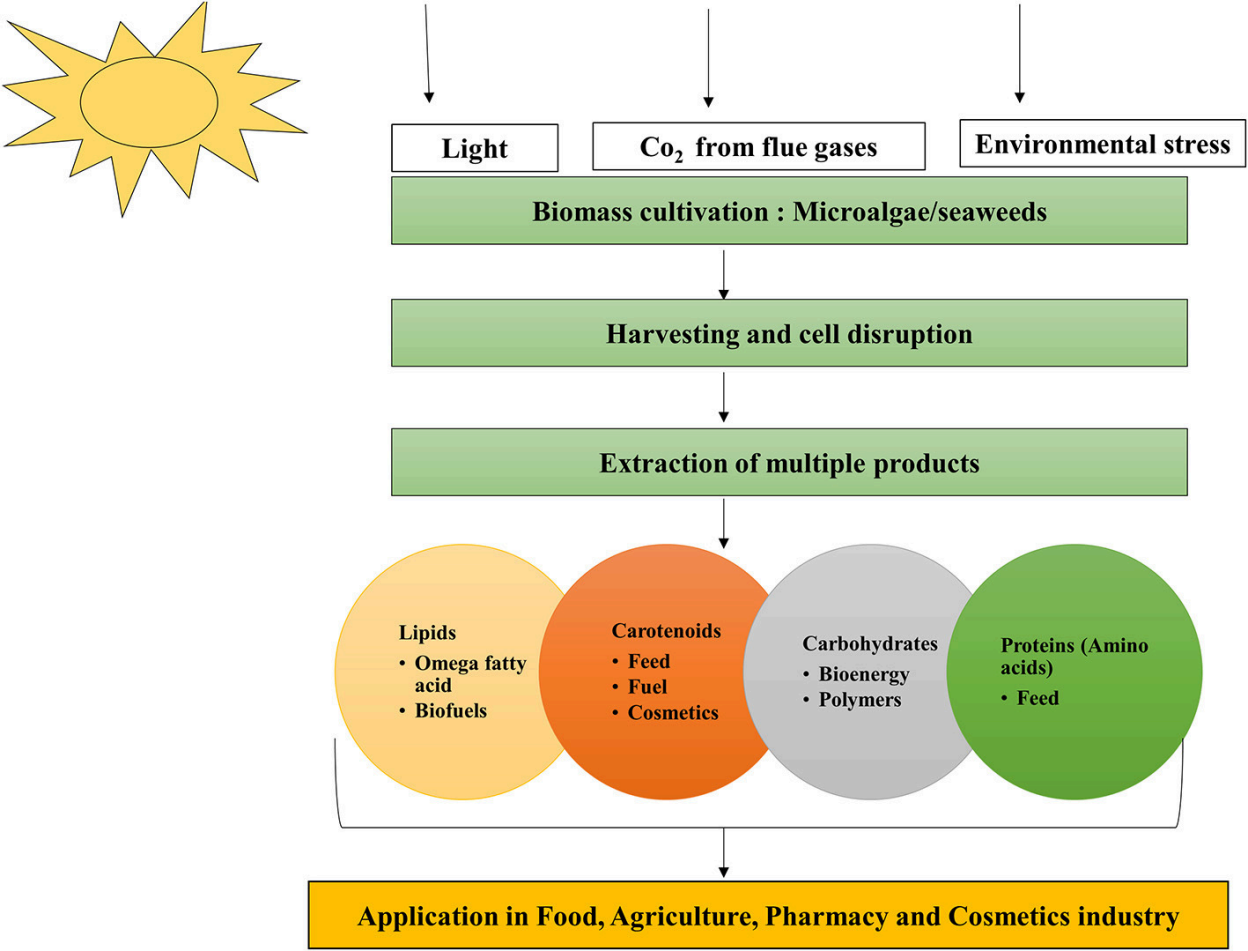
**Microalgae the biorefinery concept**.

Algae can produce a variety of antioxidants and pigments (carotenoids including fucoxanthin, lutein, β-carotene, astaxanthin, and phycobilliproteins); LC-PUFA; and proteins (the essential amino acids methionine, threonine, and tryptophan) (Gouveia, 2014) with wide applications in food, feed, agriculture, and pharmaceutical industries (Markou and Nerantzis, 2013) (Figure 3). Microalgae are useful in aquaculture as sources of biomolecules and biomass that can improve the nutritional value of food or provide additional health benefits (Yaakob et al., 2014). However, for food and aquaculture applications in particular, low cost production is important for commercial success. The production yields of microalgal biomass is higher under heterotrophic conditions than under photoautotrophic conditions. Although, the relative cost of substrate to products with respect to energy balances is more cost intensive in heterotrophic cultivation (Chen et al., 2011) compared to photoautotrophic.

The present study shows that microalgae are too expensive as a source for biofuel, without the co-production of more valuable compounds. Table 2 gives recent estimates of the production cost of microalgal value-added products along with omega FAs. Currently, three major production systems are used for microalgal cultivation: open raceway ponds, horizontal tubular photobioreactors (PBRs), and flat-panel PBRs. Raceway ponds are much better than open ponds for cultivation of microalgae (Jacob-Lopes et al., 2014). Several semi-commercial or commercial projects using open ponds and PBRs for producing value-added products from microalgae are operational worldwide (Table 2). The omega-3 FA market was worth $690 million in 2004, and the Asian Omega-3 PUFA market was expected to be worth $596.6 million in 2012 (Seambiotic, 2013). The global market for EPA and DHA is estimated at $300 million and $1.5 billion, respectively, (Table 2). A few industries in Europe and USA have also started producing EPA and DHA from microalgae (Table 2), to be used as food additives. In near future, the market for PUFA particularly omega-3) is expected to grow. PUFA [particularly, γ-linolenic acid (GLA, 18:3 ω-6), (EPA, 20:5 ω-3), arachidonic acid (ARA, 20:6 ω-6), docosapentaenoic acid (22:5 ω-3), and (DHA, 22:6 ω-3)] is gaining interest because of their application in health industry (Fraeye et al., 2012). Table 2 provides an overview of the main products from microalgae that are in the early stage of development (particularly lutein), notably for the cosmetics industry. Cyanobacteria strains produce various intracellular and extracellular metabolites that show antibacterial, antifungal, antiviral, antitumor, anti-HIV, anti-inflammatory, antioxidant, antimalarial, or herbicidal properties (Semary, 2012).

One of the major producers of *Spirulina*, Hainan Simai Pharmacy Co. (China), produces 3000 tonnes of biomass annually (Priyadarshani and Rath, 2012). Table 2 shows that a large group of industries produce β-carotene, a food additive, from *D. salina* in Australia. Many species produce high concentrations of β-carotene, astaxanthin, and canthaxanthin, which have significant applications as antioxidants and natural dyes (Table 2). The market cost of β-carotene estimated at $261 million by 2010 as shown in Table 2 and expected to grow up to $334 in 2018. On the other hand, the total market value of cyanobacteria and phycobiliproteins is estimated at $6–$11 million (Yaakob et al., 2014). The commercial market for lutein is $150 million a year in USA (Fernández-Sevilla et al., 2010) and was $233 million in 2010 in the poultry industry and as a nutritional supplement (Borowitzka, 2013) which is expected to grow up to $309 by 2018. The pigment phycocyanin is produced from *Spirulina platensis* by a small number of industries and even lutein and astaxanthin are produced by many industries worldwide (Table 2). Recent trends show that industries are entering the markets for EPA, DHA, astaxanthin, and phycocyanin. Products that are well-studied include fucoxanthin, proteins, β-glucan (a polysaccharide) and phycoerithrin (a pigment).

## Combined production of lipids and carotenoids

Besides lipids having biofuel properties, various high- value added products such as vitamins, polysaccharides, phycobilins, antioxidant can also be extracted from same biomass which could be used as commodities in several biotechnology industries (Mata et al., 2010). For example, polysaccharides which are produced by several microalgal species has potential use in health industry (Markou and Nerantzis, 2013). The combined production of lipids and carotenoids from the same microalgal biomass therefore seems promising (Table 1). Members of *Chlorella* sp. Well-known for lipids production, being now used in cosmetic industry for their protein extracts (Stolz and Obermayer, 2005). Furthermore, *C. vulgaris* extract is used in antiaging creams and tissue regeneration (Spolaore et al., 2006). Data from the literature shows as depicted in Table 1 that lipid synthesis is dependent upon carotenoids accumulation (Zhekisheva et al., 2005) when exposed to high light. Fatty acid production is linked to carotenoids accumulation (Zhekisheva et al., 2002; Cerón et al., 2007). In *H. pluvialis*, carotenoids production is dependent upon TAGs production. However, astaxanthin accumulation within these TAGs bodies involves the transfer of intermediates from the early s teps of the biosynthetic pathway in chloroplasts (Grünewald et al., 2001). Under high light intensity, *Haematococcus* sp. accumulates astaxanthin within its cells (Boussiba and Vonshak, 1991), overproducing primary carotenoids (β-carotene). In addition to astaxanthin accumulation, *Haematococcus pluvialis* (Zhekisheva et al., 2005) and *Scenedesmus* sp. (Peng et al., 2012) have the potential to produce multiple biomolecules at the same time (Table 1). However, a combination of several stress factors may results in the enhanced production of carotenoids and lipids from the same biomass. For instance, in *D.salina* combined the use of light stress apparently increased the production of β-carotene (Table 1; Lamers et al., 2010). Another study conducted by Peng et al. (2012) showed accumulation of lipids and carotenoids under salinity and light stress. The emerging value of a few carotenoids (especially lutein) in the prevention and treatment of disorders, such as degenerative diseases, and of omega FAs (EPA and DHA) as a general wellness product, has strengthened the biorefinery model.

Despite the promising cultivation conditions for production of multiple metabolites from microalgae, the process has not been yet commercialized due to lack of reactors and methods that can produce large amount to supply to market. In some cases, because of limited production of biomass under stress, the overall productivity of compounds is decreased (Adams et al., 2013). However, such negative effect could be countered by applying a combination of stress factors along with the two-stage strategy employed in a given life cycle in photoautotrophic, heterotrophic, or mixotrophic condition. High light, nitrogen deficiency and high salinity could influence the production of lipids and carotenoids within one life cycle if biphasic strategies are followed with attenuated physio-chemical factors employed. It is clear that different strains of microalgae respond differently to several environmental stress factors (Table 1). Based on the literature reviewed it is understood that nitrogen deficiency will results in accumulation of higher lipid content where as salinity impacts positively the fatty acid profile and carotenoids production in diverse microalgal species.

Simultaneous production of lipids, biomass, and carotenoids may be achieved through two phase process where growth medium rich in nitrogen and phosphates in first phase for attaining higher biomass and in second phase post late log stage high salinity and high light intensity can further enhance the production of net lipids and carotenoids. The adoption of above strategies in closed systems could achieve economically sustainable process. These strategies can increase the total productivity of the metabolites and lipids in the same biomass obtained at the end of growth cycle. The exact combination of different stress factors may vary with the species drawn from different climatic conditions. To date this concept of simultaneous production of lipids and carotenoids from one life cycle and two stage process by employing several rate limiting factors have not been fully explored and needs to be pursued emphatically in future.

## Conclusions

Recent advances in biotechnology have created opportunities for the production of high value added metabolites with unique benefits in various industrial sectors. The laboratory scale microalgal cultivation elaborated within this review provides insight into the feasibility of carrying out autotrophic and heterotrophic processes under environmental stresses. Microalgae cultivation alone under heterotrophic conditions could not sustain biofuel and carotenoid production with current cultivation strategies. It is predicted that adoption of heterotrophy alone for microalgal growth is very problematic and cost intensive. Perhaps, combining heterotrophic-autotrophic two stage cultivation of microalgae will yield a product that would make the process economically sustainable. The cultivation conditions outlined are based on the experimental data of known microalgae but in principle could also be applicable for the developing strains by use of genetic engineering.

This study defines many favorable stress factors those influence the production of lipids and carotenoids. Microalgae, under optimal conditions, demonstrate a significant potential to produce multiple metabolites, many of which can be produced simultaneously in one growth cycle. Exploring biodiversity and selecting potential strains that produce the desired multiple products with maximum efficiency can pave the way to implement the biorefinery approach cost-effectively. Genetic manipulation of promising organisms can lead to higher commercial returns from the strains chosen on the basis of their physiological performance. The biorefinery approach provides new insights into the feasibility of cost-effective and continuous production of lipids and bioactive compounds from one growth cycle and possibly the two-stage cultivation process under altered physico-chemical conditions. The advantage of using culturable biological resources instead of refining the finite stocks of crude oil is that the processes will only get better with time and will lead to better economics and efficiency in tune with the growing human needs.

Microalgal biorefinery process is still in the early stage development and more focus is needed about extraction and purification of multiple products besides lipids and carotenoids from the same biomass. Therefore, more and consistent research efforts are needed to understand the trade-offs between the lipid and carotenoids production and identifying most useful metabolic engineering strategies under optimized conditions to develop better equipped biorefinery approaches. Hopefully, this review will provide a new insights in development of different cultivation strategies for enhancement of biomass rich in lipids and carotenoids using combination of stress factors in diverse microalgal strains.

## Author contributions

Manuscript preparation: AM and AA. Manuscript improvement: AA, CB, Bhavdish N. Johri, Yateendra Joshi, Shivani Srivastava and PH.

### Conflict of interest statement

The authors declare that the research was conducted in the absence of any commercial or financial relationships that could be construed as a potential conflict of interest.
